# Task-dependent alteration of beta-band intermuscular coherence is associated with ipsilateral corticospinal tract excitability

**DOI:** 10.3389/fspor.2023.1177004

**Published:** 2023-07-28

**Authors:** Na-hyeon Ko, Christopher M. Laine, Francisco J. Valero-Cuevas

**Affiliations:** ^1^ Department of Physical Therapy, California State University, Fresno, CA, United States; ^2^ Division of Occupational Science and Occupational Therapy, University of Southern California, Los Angeles, CA, United States; ^3^ Brain Body Dynamics Lab, Division of Biokinesiology and Physical Therapy, Department of Biomedical Engineering, University of Southern California, Los Angeles, CA, United States

**Keywords:** beta-band coherence, ipsilateral corticospinal excitability, manual dexterity, task dependent intermuscular coherence, stroke rehabilitation

## Abstract

Beta-band (15–30 Hz) synchronization between the EMG signals of active limb muscles can serve as a non-invasive assay of corticospinal tract integrity. Tasks engaging a single limb often primarily utilize one corticospinal pathway, although bilateral neural circuits can participate in goal-directed actions involving multi-muscle coordination and utilization of feedback. Suboptimal utilization of such circuits after CNS injury can result in unintended mirror movements and activation of pathological synergies. Accordingly, it is important to understand how the actions of one limb (e.g., a less-affected limb after strokes) influence the opposite corticospinal pathway for the rehabilitation target. Certain unimanual actions decrease the excitability of the “unengaged” corticospinal tract, presumably to prevent mirror movement, but there is no direct way to predict the extent to which this will occur. In this study, we tested the hypothesis that task-dependent changes in beta-band drives to muscles of one hand will inversely correlate with changes in the opposite corticospinal tract excitability. Ten participants completed spring pinching tasks known to induce differential 15–30 Hz drive to muscles. During compressions, transcranial magnetic stimulation single pulses to the ipsilateral M1 were delivered to generate motor-evoked potentials in the unengaged hand. The task-induced changes in ipsilateral corticospinal excitability were inversely correlated with associated changes in EMG-EMG coherence of the task hand. These results demonstrate a novel connection between intermuscular coherence and the excitability of the “unengaged” corticospinal tract and provide a springboard for further mechanistic studies of unimanual tasks of varying difficulty and their effects on neural pathways relevant to rehabilitation.

## 1. Introduction

Neural drive from the motor cortex (M1) to contralateral muscles often includes beta-band (15–30 Hz) oscillations, the strength of which can serve as an index of cortical excitability and corticospinal tract integrity (1–11). Corticomuscular drive within the beta-band is strongly associated with the excitability of the associated corticospinal tract during unimanual, isometric finger abduction, and wrist extension tasks (12, 13) or isometric tibialis anterior contraction (14). The strength of this oscillatory component of the cortical drive must be interpreted with careful attention to task conditions as it depends on multiple functional factors such as muscle coordination, force magnitude, and limb movement (2, 15–19).

Moreover, while studies mostly focus on how actions of one hand/limb correspond with the beta-band activity and/or excitability of the contralateral cortex (12, 15, 20), unimanual tasks can involve both hemispheres (21–29). Unimanual tasks with, say, the right hand can also alter the excitability of the corticospinal pathway originating ipsilateral to the task hand (21–23, 30–33)—that is, the ipsilateral corticospinal tract from the right brain to the left hand. Highly dexterous unimanual tasks have been shown to activate a multitude of brain areas, including the bilateral primary motor cortex, ventral premotor cortex, posterior parietal cortex, basal ganglia, cerebellum, etc., presumably due to the difficulty and dynamics of the required sensorimotor integration (25–28).

This is a critical consideration after stroke, where beta-band cortical activity becomes synchronized with ipsilateral muscles (3, 5, 34, 35), where recovery and prevention of mirror activity may depend on balanced excitability between cortical hemispheres (36, 37), and where inappropriate recruitment of compensatory circuits (e.g., reticulospinal pathways) may generate pathological synergies (38–41).

In this light, unimanual tasks may not only serve as a simple testbed for mechanistic studies but, by virtue of engaging multiple bilateral neural circuits depending on task difficulty, may contribute to clinical applications such as using a less-affected limb to assist in the neurorehabilitation of a more-affected limb after stroke. Development in this area would require that the relevant neural effects of particular tasks can be predicted and monitored.

In this study, we tested the hypothesis that task-related changes in the strength of beta-band corticomuscular drive to muscles of a task-engaged hand would inversely correlate with changes in the excitability of the unengaged (ipsilateral) corticospinal tract. Previous studies have shown that changing the dexterity demands of pinching tasks can alter beta-band drive to contralateral muscles (15), and fine sensorimotor tasks can alter the excitability of the ipsilateral corticospinal tract (33). While it is possible that both phenomena are related, perhaps reflecting interhemispheric balance, they have not been examined together, and thus their potential correlation is unknown.

## 2. Methods

Ten healthy adults (29.5 ± 3.5 years, 4M, 6F) participated in the study. All participants were right-handed (self-reported). They had no history of neurological or musculoskeletal disorders or surgeries and no ongoing pain in the thumb and index finger at the experimental session. All participants were screened for TMS eligibility using a TMS safety questionnaire. Each provided written informed consent, and the study protocol was approved by the Institutional Review Board at the University of Southern California.

### 2.1. Motor task

The participants completed two precision pinch tasks during which beta-band EMG-EMG coherence was measured between the first dorsal interosseous (FDI) and abductor pollicis brevis (APB) muscles. The first task was the compression of a spring *less* prone to buckling when compressed (i.e., “stable” spring, easy), which produces a strong beta-band corticospinal drive to the FDI and APB muscles (15). The second task was a force-matched compression of a spring *more* prone to buckling when compressed (i.e., “unstable” spring, difficult) (15, 42–46). These springs (Valero Dexterity Test®, Neuromuscular Dynamics, LLC, La Crescenta, CA) were custom-designed with the same spring constant but with different lengths (42, 46). The unstable spring is longer and thus prone to buckling and is challenging to compress fully, requiring the greatest dexterity demands of dynamic control of fingertip force vectors (magnitudes and directions) at low forces (<3N) (42, 46). The unstable spring would buckle without continual dynamic adjustments, and we have shown that this task reduces corticomuscular and intermuscular coherence at beta-band frequencies (15). In contrast, the stable spring is shorter and thus more easily compressed to the desired force level and requires relatively low dexterity demands (42). The target force level for both springs was set per individual as 95% of the force that could be consistently exerted on the unstable spring and held for 7 s without it buckling. Forces were acquired from the spring using a miniature load cell (ELB4-10, Measurement Specialties, Hampton, VA, USA) connected to a USB-data acquisition unit (National Instruments, Austin, TX, USA). The visual feedback was provided using custom MATLAB (MathWorks, Natick, MA, USA) scripts. Each participant performed 17–25 pinches with each spring. Each pinch consisted of a 1-second ramp-up phase, a hold phase of the target force level for 7 s, and a 1-second ramp-down phase. Practice trials were provided prior to recordings to ensure the tasks could be completed consistently without error.

### 2.2. EMG recordings

To measure beta-band (15–30 Hz) neural drive to the engaged muscles, EMG signals were recorded from the first dorsal interosseous (FDI) and abductor pollicis brevis (APB) muscles of the right hand. Active surface EMG sensors (Motion Lab Systems, Inc., Baton Rouge, LA, USA) amplified and bandpass filtered the EMG signals at 15–3,500 Hz. The EMG data were acquired at 14,993 Hz and collected using the CED 1401 interface unit and associated Signal 2 software (Cambridge Electronic Design, UK). EMG was also recorded from the resting FDI to monitor incidental activity that might influence TMS measurements.

### 2.3. TMS protocols

**Figure 1:**
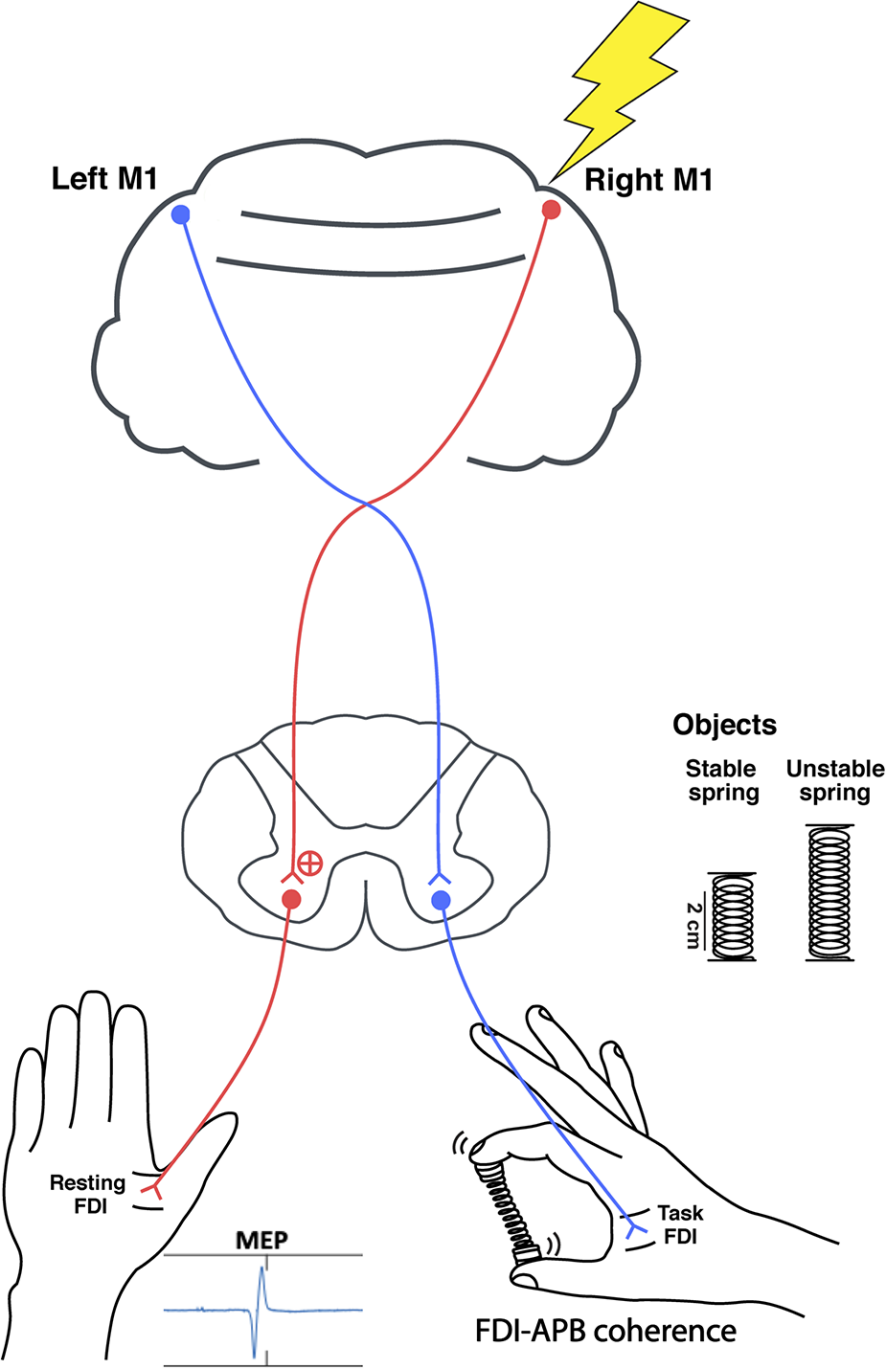
The study set up. Single pulse TMS was delivered over the right M1 during the spring hold phase while compressing either a stable or unstable spring. Peak-to-peak MEPs were recorded from the resting left FDI, and right FDI-APB muscle coherence was quantified.

To measure the corticospinal excitability of the ipsilateral M1 during the hold phase of the unimanual pinch tasks, single pulses of TMS (Magstim 200; Magstim Company Ltd., Whitland, UK) were delivered over the right M1 representational area of the first dorsal interosseous (FDI) of the resting hand (Figure 1). Participants sat comfortably with the right forearm supported with a foam cushion. Their left arm and hand rested comfortably and were supported by pillows. A figure of eight coil (70 mm diameter) was placed tangentially with the handle pointing backward and laterally 45 degrees from the midline so that the induced electric current flowed in a posterior to anterior direction (47). The coil was initially placed 5 cm laterally, and 2 cm anteriorly from the vertex, and moved by 1 cm increments on a Lycra cap fitted to the subject while searching for the motor hot spot, which was defined as the location producing the largest amplitude and most consistent motor evoked potentials (MEPs). The resting motor threshold (RMT) was then determined as the minimum intensity that induced a peak MEP greater than 50 μV for 5 out of 10 trials. Finally, single pulses were delivered over the hot spot of the ipsilateral, right M1 at 120% of the RMT during the hold phase. A total of 20 trials were collected per condition. The timing was varied to prevent anticipation of the pulse.

### 2.4. Data analysis

To quantify ipsilateral corticospinal excitability, the average peak-to-peak MEPs of FDI was calculated per task, per individual. To quantify the extent of beta-band drive to the task-engaged hand, the 1s epochs of EMG from the right FDI and right APB that preceded each TMS pulse were concatenated per individual and used to calculate pooled coherence (48), using the MATLAB's mscohere function, specifying Gaussian tapering of 500 ms windows, overlapped by 80%. To account for small variation in the number of compressions completed per task and facilitate comparisons across participants, raw coherence was converted to standard *Z*-scores using the formula $z\;=\frac{atanh\left(\sqrt{c}\;\right)}{\surd (1/2L)}\;-\;\mathrm{bias}$, where *L* is the number of segments used in the coherence analysis [with overlap accounted for as in (49)] and the bias calculated empirically as the mean *z*-value between 100 and 300 Hz (50–52). The average coherence within the 15–30 Hz frequency range was calculated per task, per individual.

The cross-task difference in MEP size and 15–30 Hz coherence were calculated for each participant and then tested for correlation (Spearman's rho). Spearman's correlations were also calculated for each measure (MEP size and coherence) across subjects within each task. To determine if task-related differences in unintentional left (resting) FDI activity might have influenced MEP measurements, the percent change in average EMG amplitude across tasks was tested for correlation with MEP size across subjects. Similarly, the percent changes in task-engaged muscle activity (right FDI and APB) were tested for correlation with the changes in 15–30 Hz coherence across tasks, to investigate possible influence of activity-associated signal-to-noise ratios or cross-talk. Percent changes in compression force along the axis of the spring as well as in compression force variability (as a metric of task performance) were calculated as well and tested for correlation with task-associated changes in MEP size and coherence. Signed-rank tests were used to test the significance of changes in MEP amplitudes and coherence across tasks.

## 3. Results

### 3.1. Corticospinal excitability

**Figure 2:**
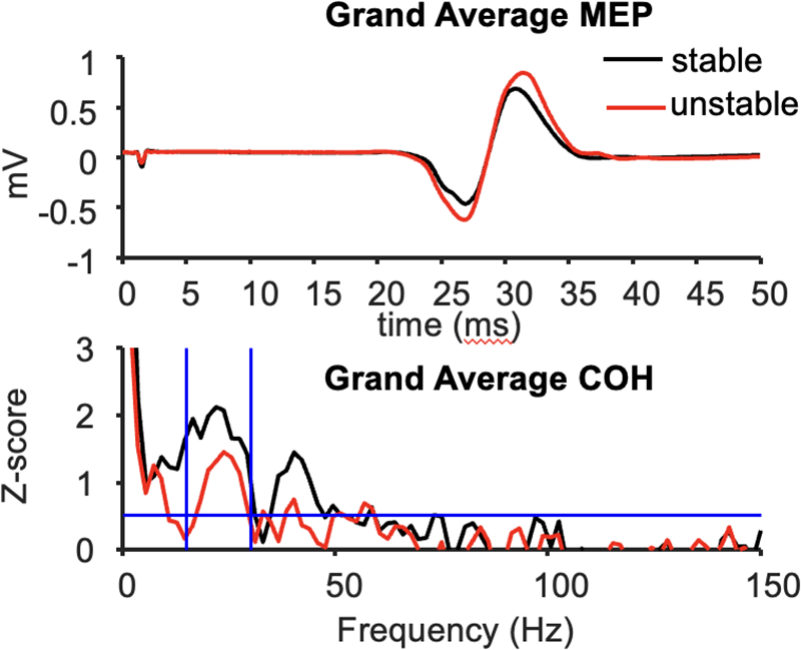
Grand average MEP waveforms (top) and FDI-APB intermuscular coherence (bottom) across 10 subjects.

**Figure 3:**
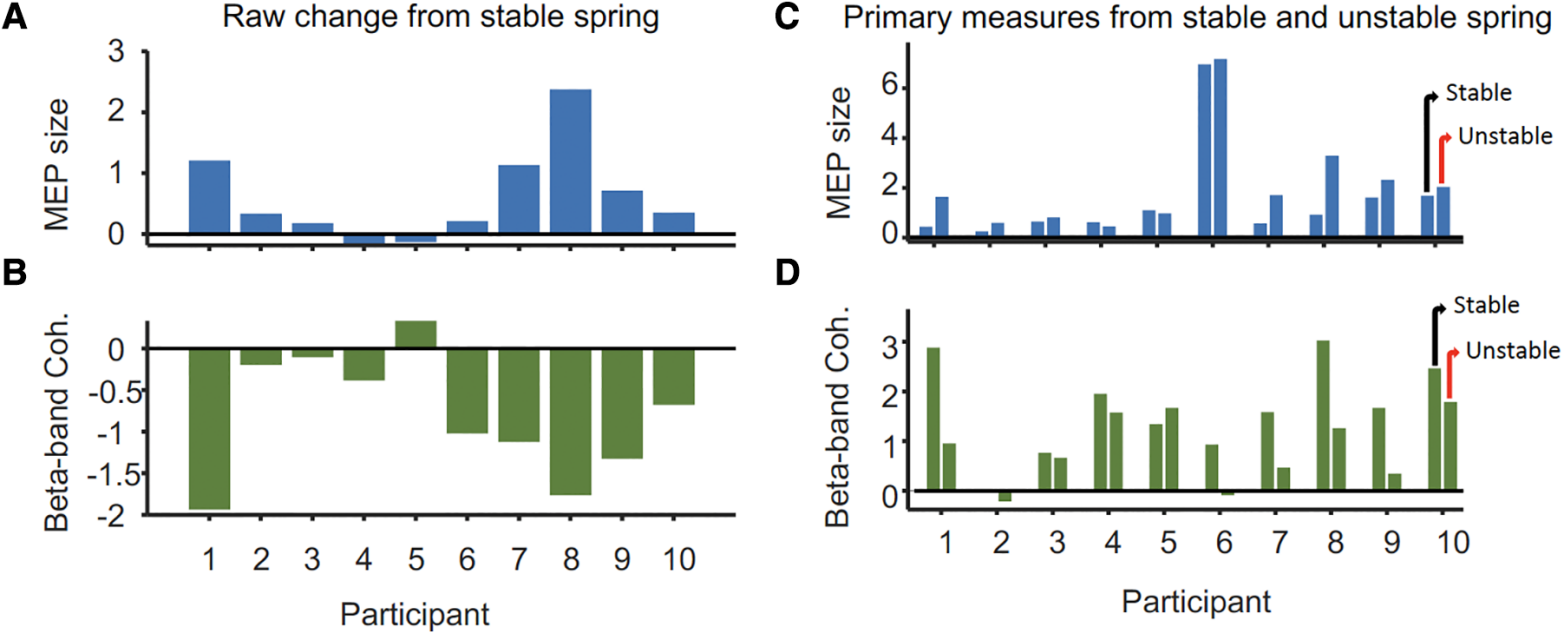
Differences in (**A**) peak-to-peak MEP size (mV) from the ipsilateral M1 and (**B**) 15–30 Hz FDI-APB intermuscular coherence (mean *Z*-score) of the task hand between stable and unstable spring tasks per participant. Panels (**C,D**) show the MEP and coherence values from which the changes shown in (**A,B**) were derived. For each participant, values for the unstable spring compression are on the right and stable on the left.

The mean peak-to-peak MEP amplitude in the resting left FDI was significantly larger during compression of the unstable vs. stable spring (*p* = 0.014, Figure 2), and this tendency was directionally consistent for 8/10 individuals (Figure 3A). Figure 3C shows the MEP values per individual in each task.

### 3.2. Beta-band drive to task-engaged muscles

In contrast, beta-band FDI-APB coherence was significantly smaller during compression of the unstable vs. stable spring (*p* = 0.0098), and this effect was directionally consistent for 9/10 individuals (Figure 3B). Figure 3D shows the coherence values per individual and task.

### 3.3. Correlation between MEP size and EMG-EMG coherence

**Figure 4:**
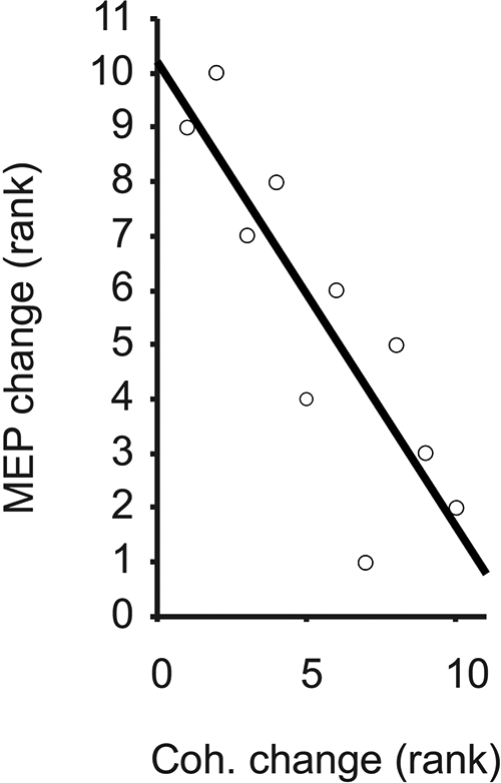
Ranked change in MEP vs. FDI-APB coherence, with trend line illustrating Spearman's rank correlation (rho = −0.84).

Task-related changes in MEP size were significantly and inversely correlated with the task-related changes in beta-band coherence across the 10 individuals (rho = −0.84, *p *= 0.0045, Figure 4). Given the individual MEP and correlation measurements as shown in Figures 3, 4, it appears the relationship is not only monotonic but reasonably linear (Pearson's rho = −0.81), although modeling the precise relationship is beyond the scope of this study. Within each task, the amplitudes of MEPs across individuals were not correlated with the strength of beta-band coherence (rho = 0.09 and 0.22 for stable and unstable spring compressions, respectively).

The mean (±SD) cross-participant percent (%) difference in compression force during unstable spring compression relative to stable spring compression was −3 (±7) %, and these changes did not correlate with changes in MEP amplitudes (rho = 0.03), or 15–30 Hz coherence (rho = 0.03). Likewise, the mean (±SD) change in compression force variability relative to stable spring compression was −1 (±50) %, and these changes did not correlate with changes in MEP size (rho = −0.09), or coherence (rho = 0.43) across participants. The mean (±SD) amplitude of right FDI activity was 126 (±223) % greater in the unstable vs. stable spring condition, and for the APB muscle, the change was 60 (±82) %. However, the correlation between changes in FDI or APB amplitudes and changes in coherence were not statistically significant (rho = −0.61 and −0.64, respectively). The resting (left) FDI EMG signals were 46 (±61) % larger during unstable spring compression compared with the stable spring condition, but again, these changes did not correlate significantly with task-related changes in MEP amplitudes (rho = −0.47). In summary, task-associated changes in 15–30 Hz coherence and MEP sizes were strongly, and significantly correlated with each other across participants, but they were not significantly correlated with other measures of task performance or physical effort.

## 4. Discussion

The study demonstrates a novel relationship between task-related changes in beta-band intermuscular coherence of an *engaged* hand and corticospinal excitability of the *unengaged* hand. These findings emphasize that the corticospinal excitability of either hemisphere can be manipulated by the difficulty of the task performed by either hand, and further, that the extent of this effect may be measurable using a simple, passive measurement of EMG signals (i.e., intermuscular coherence).

In general, movement and dynamic actions reduce beta-band neural drive from M1 to muscles (15, 53), but the extent of this reduction from one task to another has not been directly related to associated changes in corticospinal excitability, especially from the unengaged M1. Rather, corticospinal excitability has been manipulated directly via transcranial direct current stimulation (tDCS) (12) and beta-band oscillatory tDCS (14). The consequence of cathodal tDCS was a reduction of beta-band coherence among muscles during an isometric muscle contraction, along with a correlated decrease of MEP amplitudes evoked from the contralateral M1 (12). Our study shows that this basic relationship between the beta-band drive to muscles and corticospinal excitability also holds (albeit with reversed direction) when a physical task is used to change neural activity rather than tDCS. However, our novel observation of an inverted relationship between intermuscular 15–30 Hz drive, originating in the contralateral cortex and ipsilateral corticospinal tract excitability, may suggest that excitability is balanced across hemispheres during our dexterous tasks. Thus, a change in the excitability of one corticospinal tract (indexed by beta-band intermuscular coherence) is matched by an opposite change in the corticospinal excitability of the opposite hemisphere ipsilateral to the engaged hand (measured via MEP amplitudes).

Of course, without more sophisticated approaches (e.g., testing for intracortical excitability, interhemispheric inhibition, stretch reflex modulation, etc.), we cannot identify specific neural mechanisms that might underlie the observed effects. It is also worth noting that in this study, 15–30 Hz intermuscular coherence can only be assumed to represent a cortical drive since cortical EEG was not measured. However, decades of investigation characterizing beta-band drive to muscles in terms of synchrony between motor unit spike trains, between surface EMG signals, or between EEG and EMG signals has consistently pointed to this frequency band representing a cortical drive (1, 7, 15, 54–56). In fact, when we have monitored both corticomuscular and intermuscular coherence together during tasks similar to that of the present study (15), the two measures correspond closely and have similar task-dependencies. Thus, we consider it appropriate to utilize, for the sake of speculation and interpretation, a combination of studies which may discuss beta-band drive to muscles using different measurement methods.

Previous single-pulse TMS studies have shown task-dependent modulation of the ipsilateral corticospinal excitability (22, 23, 57) during unimanual tasks. Larger unimanual finger forces and overt movement (e.g., finger opposition sequence tasks or rhythmic index finger abduction) (23, 57) increase corticospinal excitability of the resting hand compared with lower force, static tasks (21, 23, 30–32). However, these effects likely do not explain our results, where pinch forces were very low (<3N), and finger movements were necessarily small in order to prevent the spring from buckling (42). The reason might be the required motor control strategy and the involvement of different neural structures for such dexterous tasks (25–28). Controlling the instability of the unstable spring emphasizes ongoing dynamic corrective responses to tactile/proprioceptive feedback as opposed to purely feed-forward planning of predetermined actions (58). Although we did not quantify total co-contraction of all active hand/wrist/forearm muscles, or monitor subtle changes in fingertip position in space during each pinch, neither feature of muscle output provides a clear connection between beta-band coherence and corticospinal excitability. In fact, task-associated changes in muscle activity in either hand did not correlate highly with changes in MEP sizes or coherence, suggesting that task-related differences in overall drive to the muscles, and associated issues of cross-talk and signal to noise ratios, are very unlikely to explain our main findings. That said, lack of statistical significance should not be taken as evidence of zero influence, since our sample size allows reliable detection of only very strong effects. Larger studies, perhaps with single motor unit recordings and a larger set of recorded hand/finger muscles, would be needed to fully characterize the extent to which task-associated changes in muscle activation might have influenced our measures. Ultimately, our results suggest that the more salient difference between the stable and unstable spring was the change in brain-wide neural motor control requirements rather than simply a required change in physical forces/movement.

Exactly which neural circuits became more engaged when controlling the unstable spring, and how these circuits impacted our measures will require further research to understand. Tasks requiring quick movement corrections involve desynchronization of the beta band corticomuscular coherence (15), which might result from the decreased activity of the inhibitory interneurons in the sensorimotor cortex (53) and require multiple brain areas and subcortical structures to execute the task successfully (25–28). The spring task used in this study involves the bilateral cortico-striatal-cerebellar network, which is modulated by the degree of instability (26). Greater bilateral activity in the basal ganglia (BG) is associated with greater instability of the spring (26). Neuroanatomically and functionally, the bilateral BG is connected to M1, forming a sensorimotor cortico-striatal loop (59–61), which might influence the ipsilateral M1 excitability (29). The increased blood-oxygen-level-dependent (BOLD) signal from the fMRI study does not elucidate if this was because of excitatory or inhibitory neural mechanisms. However, increased excitatory neural drives within the bilateral cortico-striatal-cerebellar network are likely to result in ipsilateral M1 excitability, perhaps explaining our results. However, to identify specific underlying neural mechanisms of communication between two hemispheres, further research, including studies of interhemispheric inhibition, will be necessary.

Our observations may ultimately stem from the activation of subcortical circuits to meet increased demands on sensorimotor integration and muscle control, consequently altering cortical oscillations and excitability. If so, it would be relevant for neurorehabilitation efforts to explore the possibility that a unimanual task can access fundamental (and bilateral) neural control circuits at and across different hierarchies of the central nervous system. For instance, a unimanual task with such sensorimotor control requirements could possibly be used for priming the affected neural circuits before or during motor rehabilitation in individuals with stroke instead of using repetitive TMS. In addition, task-based neuromodulation or re-education of functional neural circuits may be feasible in individuals with Parkinson's disease for implicit, reactive motor control, considering the involved neural circuits and their ultimate effect on motor impairment (62). Furthermore, the desired effects can be imposed and modulated by the difficulty of physical tasks. Any such effort to alter excitability or prime a particular circuit may be measured peripherally (e.g., via EMG coherence) or centrally (via brain network analyses) if our initial results generalize to these clinical contexts.

Future work is required to determine whether task-associated changes in ipsilateral corticospinal excitability necessarily involve alteration of contralateral beta-band activity and the extent to which altered intermuscular coherence can be used as a simple, non-invasive predictor of this effect. Our findings serve as a springboard for detailed mechanistic work along these lines, as well as an unambiguous demonstration of the connections between tasks, different frequencies of neuromuscular drive, and corticospinal excitability. This may have clinical value in the assessment of neuropathology or clinical application to neurorehabilitation.

## Data Availability

The raw data supporting the conclusions of this article will be made available by the authors, without undue reservation.
